# Effectiveness of bystander naloxone administration and overdose education programs: a meta-analysis

**DOI:** 10.1186/s40621-015-0041-8

**Published:** 2015-05-22

**Authors:** Rebecca E Giglio, Guohua Li, Charles J DiMaggio

**Affiliations:** 1 Center for Injury Epidemiology and Prevention, Columbia University, 722 West 168th St, 5th Floor, New York, 10032 NY USA; 2 Department of Epidemiology, Columbia University Mailman School of Public Health, 722 West 168th St, New York, 10032 NY USA; 3 Department of Anesthesiology, Columbia University College of Physicians and Surgeons, 622 West 168th St, PH5-505, New York, 10032 NY USA; 4 Department of Surgery, New York University School of Medicine, 550, First Avenue, New York, NY 10016 USA

**Keywords:** Opioid overdose, Naloxone, Overdose prevention, Bystander

## Abstract

The objective of this review was to assess the effectiveness of bystander naloxone administration and overdose education programs by synthesizing quantitative results reported in the research literature. Studies meeting predefined criteria were identified and reviewed, and their results were synthesized through meta-analysis. Odds ratios (ORs) and 95% confidence intervals (CIs) were calculated for overdose recoveries for individuals who received naloxone dispensed by non-medical community members, and the standardized mean difference was calculated for test scores of non-medical volunteers who received training in overdose management versus the scores of untrained volunteers. Pooled data from four studies showed that naloxone administration by bystanders was associated with a significantly increased odds of recovery compared with no naloxone administration (OR = 8.58, 95% CI = 3.90 to 13.25). Data from five studies of overdose education indicated that average scores were significantly higher for trained participants than untrained participants for tests on naloxone administration, overdose recognition, and overdose response (standardized mean difference = 1.35, 95% CI = 0.92 to 1.77). Empirical evidence in the research literature suggests that bystander naloxone administration and overdose education programs are associated with increased odds of recovery and with improved knowledge of overdose recognition and management in non-clinical settings.

**Electronic supplementary material:**

The online version of this article (doi:10.1186/s40621-015-0041-8) contains supplementary material, which is available to authorized users.

## Review

### Introduction

The population-based death rate from drug overdose in the United States has increased dramatically over the last two decades, primarily due to an increase in deaths involving opioid analgesics (CDC 2014a,b,c). In 2013 (the last year for which data are available), national data indicate that drug overdose claimed 43,982 lives, 81.1% of which were coded as unintentional (CDC. National vital statistics system mortality data 2015). Overdoses involving opioid analgesics accounted for 36.9% of all drug-poisoning deaths (CDC. National vital statistics system mortality data 2015).

Opioid overdose deaths are avoidable if the antagonist naloxone is administered in time. Naloxone has been the standard treatment for overdose effects like respiratory depression, sedation, and hypotension in the emergency department setting for the past 3.5 decades (Kim et al. 2009). In 1996, community-based overdose prevention programs began distributing naloxone to high risk opioid users (CDC 2012).

There is a general consensus in the literature on the safety and effectiveness of naloxone when administered by health professionals (Sporer 1999). Naloxone has few known adverse effects, has no potential for abuse, and is available at a reasonably low cost (Maxwell et al. 2006), although there has been recent concern over cost increases. Some states have experienced increases of more than 50%. For example, the price of a naloxone kit in Georgia, originally $22, has spiked to $40 (Goodman 2014).

The effectiveness of naloxone is entirely time dependent. Death typically occurs within 1 to 3 hours after an overdose (Kim et al. 2009). Thus, naloxone is only successful in reversing an overdose if administered before overdose symptoms cause death. Medical first responders and emergency departments are equipped with naloxone. However, it is often the case that these service providers arrive too late to revive overdose victims.

Bystanders may be reluctant to call 911 because of fear of police involvement (in many communities, police respond to 911 calls with EMS) (Kim et al. 2009). Even when a bystander’s call is immediate, transportation time adds delays. These time costs may be avoided when naloxone is available at the scene of the overdose, for example, in the hands of drug users and their social networks. Harm reduction workers have resolved to equip opioid users and their community with the antidote (Kim et al. 2009).

Since their introduction, naloxone distribution programs have prompted both ethical and legal concerns. Some critics have argued that possession of naloxone could increase reckless drug use, if users considered naloxone a ‘safety net’ against the fatal consequences of use (Seal et al. 2003). Research evidence generally contradicts this claim, as studies have found that participants in naloxone programs report decreased use at follow-up and/or an intention to actively avoid the risk of overdose in the future (Seal et al. 2005, Bigg 2002). Critics have also questioned whether or not drug users and community members can be trusted to safely and adequately respond to overdoses.

This study presents a systematic review of the literature on bystander and non-medical administration of naloxone, synthesizes the effect estimates of studies reporting quantitative outcomes, and reports on the effectiveness of naloxone administration by bystanders in reversing overdoses as well as whether overdose response training increases knowledge of overdose recognition and management. The objective of this study is to synthesize the quantitative findings of available studies to generate a summary estimate of the effectiveness of such programs using meta-analytic methods.

### Methods

This study followed the guidelines for conducting systematic reviews and meta-analyses of observational epidemiological studies, as outlined in the meta-analysis of observational studies in epidemiology (MOOSE) guidelines (Stroup et al. 2000).

#### Search and ICD-10 coding

We electronically searched PubMed and additional sources for published studies using the following search terms: use*, using, addict*, disorder*, naloxon*, narcan*, evizo, OEND, OOPP, THN, overdose, overdos*, educat*, train*, untrain*, un-train*, nontrain*, non-train*, and program*. The articles were entered into the Endnote bibliographic management software program (Reuters, 2013). Abstracts and titles were electronically searched in Endnote for the terms naloxone, opioid antagonist, overdose, distribution, and program. Visual inspection identified animal studies, pharmacological studies, and treatment program studies for exclusion. Full-text versions were reviewed by the primary author and coded for naloxone recovery and death rates, as well as the following training variables: naloxone administration knowledge, overdose response knowledge, and overdose recognition knowledge.

Inclusion criteria consisted of studies quantitatively measuring the impact of overdose prevention program training sessions on the knowledge of community members. Training material had to cover, at minimum, naloxone administration, for example, the intranasal administration technique and other overdose response strategies, such as calling 911 or using rescue breathing. Eligible studies compared individuals who had been trained in prevention programs to individuals who had not yet been trained in a program at the time of their participation in the study. Eligible studies clearly defined their participant population.

Articles on naloxone distribution programs were excluded from this analysis if they did not distinguish between naloxone administration by emergency personnel and naloxone administration by lay people or did not report one of the following outcomes: recoveries with naloxone, recoveries without naloxone, deaths with naloxone, and deaths without naloxone. Articles on overdose prevention training were excluded if they did not report a mean untrained participant test score and a mean trained participant test score or if these numbers were not deducible.

For both the analysis of overdose reversal and the analysis of training effectiveness, randomized control trials, cohort studies, or cross-sectional analyses with validated measures were included. Included studies were required to include a control group, either of untrained participants or of participants prior to training. All eligible studies reported at least one quantitative outcome measure of overdose prevention skills learned. These measures could be objective tests of knowledge or skills acquired in training or subjective reports of performance. Only studies from which an effect size could be computed were included. All studies had to be written or translated into English, in order to be comprehensible to the researchers.

Studies were included in the analyses if they reported on programs which trained lay community members in overdose management. Therefore, trained participants could be defined as substance users, family and friends of substance users, or community members with no direct relation to substance users. Trained participants could not include health care professionals, EMTs, or police officers, as this study aimed to evaluate the effectiveness of these programs in training members of the community with no medical or emergency service experience.

#### Quality appraisal

A quality appraisal was conducted to assess the methods of all studies which reported the necessary outcomes and otherwise fulfilled the inclusion criteria. The quality appraisal was adapted from the assessment of quantitative studies scale created by Jinks et al. (2011), which has been implemented by other studies (Clark et al. 2014). The articles were rated on eight items. A perfect score was an 8/8.

Implementation of the scale differed slightly from implementation by Clark et al. (2014). This was only relevant for the rating of the study by Lankenau et al. (2013). This discrepancy can be attributed to slight differences in scoring. Clark used a separate randomization criterion for qualitative studies, whereas this study evaluated both qualitative and quantitative studies using the criterion provided by Jinks et al. (2011). Additionally, Clark used a scale of 0, 0.5, and 1, while this study gave ratings of either 0 (for absent criteria) or 1 (for present criteria). The primary author of this study was blind to Clark’s ratings when assessing articles.

#### Data analysis

Studies were grouped by the outcome reported, either dichotomous (naloxone success rate) or continuous (average training score). Results were tabulated and effect sizes were calculated. For the dichotomous naloxone effectiveness outcome, odds ratios were computed based on the reported naloxone success rates. For the training effectiveness outcome, standardized mean differences were calculated based on the reported average training scores. Fixed and random effects models were developed, and statistical significance at 95% confidence intervals was computed. A random effects model was most appropriate for this analysis because of its implications for generalizability. The fixed effects model assumes that the included studies are functionally identical and therefore would only describe naloxone distribution program participants. The random effects model provides a more conservative estimate because it accounts for between-study variance. In this model, we added the following term to account for between-study variance: (*Q*-*k*-1)/SUM(*w*)-(SUM(*w*^2^)/SUM(*w*)), where *Q* is the Q statistic based on chi squared, *k* is the number of studies, and *w* is the weight for each individual study. However, since the number of studies in this analysis is small, the random effects model cannot estimate between-study variance with much precision (Borenstein et al. 2009). We included both models to show that the summary estimate is not greatly influenced by model choice.

Heterogeneity was assessed using the Q statistic by Cochran (1954) and the *I*^2^ index by Higgins and Thompson (2002). The Q statistic indicates whether heterogeneity is statistically significant whereas the *I*^2^ statistic quantifies the extent of heterogeneity. The latter reveals the proportion of variability in a meta-analysis that is the result of between-study variation and not error within studies due to random sampling. *I*^2^ ranges from 0% to 100%, higher percentages indicating substantial heterogeneity (Huedo-Medina et al. 2006).

All analyses were conducted using Comprehensive Meta-Analysis version 2 and the R statistical computing platform (Biostat Inc., Englewood, NJ, USA).

### Results

**Figure 1:**
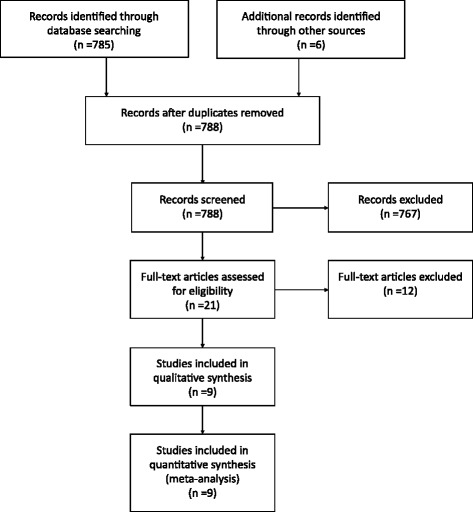
PRISMA flow diagram of identification of articles for inclusion adapted from Moher et al. (2009).

A total of 785 studies were identified through database searching, and six additional studies were identified through other sources. Of these, three duplicates were removed (see Figure 1). Because no article received a low quality rating**,** no article was eliminated from this study on the basis of its quality rating (see Table 1).

**Table 1:** **Quality ratings of included studies**

**Authors**	**Research questions/objectives/hypothesis are clear and appropriate**	**Clear overview of intervention is given with use of appropriate outcome measures**	**Sample size is given**	**Randomization method used in sample selection**	**Attrition rate is recorded and discussed**	**Data analysis is adequately described and rigorous**	**Outcomes are clearly described**	**Ethical issues are suitably addressed**	**Total score**
Williams et al. (2014)	1	1	1	1	1	1	1	1	8
Gaston et al. (2009)^a^	1	1	1	0	1	1	1	1	7
Strang et al. (2008)^a^	1	1	1	0	1	1	1	1	7
Green et al. (2008)	1	1	1	0	N/A	1	1	1	6
Jones et al. (2014)	1	1	1	0	N/A	1	1	1	6
Lankenau et al. (2013)	1	1	1	0 [0.5]	N/A	1	0 [0.5]	1	6
McAuley et al. (2010)	1	1	1	0	1	1	1	1	7
Galea et al. (2006)	1	1	1	0	1	1	1	1	7

^a^Strang et al. (2008) uses the same sample as Gaston et al. (2009); Values in brackets are ratings that differ from those attributed by Clark et al. (2014).

#### Description of included studies

Four studies contributed to the analysis on bystander naloxone administration (Strang et al. 2008; Lankenau et al. 2013; McAuley et al. 2010; Galea et al. 2006). Naloxone outcomes were defined as ‘recoveries with naloxone,’ ‘recoveries without naloxone,’ ‘deaths with naloxone,’ and ‘deaths without naloxone.’ Lay-dispensed naloxone outcomes were based on participant self-reports from witnesses of overdoses. Participants indicated who administered naloxone and whether or not the victim was revived. McAuley et al. (2010) was the only study to not rely on self-report. For all studies, self-reports were confirmed with police and ambulance data (see program characteristics in Table 2).

**Table 2:** **Characteristics of the programs in the meta-analysis on bystander naloxone administration**

**Study**	**Program site(s)**	**Subjects**	**Follow-up(s)**	**Study design**
Strang et al. 2008 ^a^	Birmingham	Users	3 months	Prospective cohort
	London			
Lankenau et al. 2013	Los Angeles	Users	NR	Cross-sectional
McAuley et al. 2010	Lanarkshire	High risk users	2 months	Cohort
			6 months	
Galea et al. 2006	New York City	Users	3 months	Cohort

^a^Strang et al. (2008) uses the same sample as Gaston et al. (2009).

Five studies contributed to the analysis on training effectiveness (Green et al. 2008; McAuley et al. 2010; Williams et al. 2014; Gaston et al. 2009; Jones et al. 2014) (see program characteristics in Table 3). Training outcomes were defined as ‘trained participant training score’ and ‘untrained participant training score.’ McAuley et al. (2010) was the only study to report both naloxone and training outcomes. Strang et al. (2008) and Gaston et al. (2009) report on the same sample at different time periods and are therefore discussed here as a single study.

**Table 3:** **Characteristics of the programs in the meta-analysis on training effectiveness**

**Study**	**Program site(s)**	**Subjects**	**Follow-up(s)**	**Study design**
Green et al. 2008	Baltimore	Users	N/A	Cross-sectional
	San Francisco			
	Chicago			
	New York			
	New Mexico			
McAuley et al. 2010	Lanarkshire	High risk users	2 months 6 months	Cohort
Williams et al. 2014	London	Family/friends of users	3 months	Randomized controlled trial
	Kent			
	Herefordshire			
Gaston et al. 2009 ^a^	Birmingham	Users	3 months 6 months	Cohort
	London			
Jones et al. 2014	New York City	Users	N/A^b^	Cohort

^a^Gaston et al. (2009) uses the same sample as Strang et al. (2008).

^b^Post-training test administered immediately after training.

Training outcomes were measured by comparing the test scores of those who had completed training with those who had not. In all but one study, the training outcome was a comparison of pre- and post-training scores completed by the same group of participants. In Green et al. (2008), the trained and untrained participants were distinct samples (the untrained participants did not go on to be trained).

All training programs briefed participants on naloxone distribution (for example, how to assemble the applicator and release naloxone) and overdose response (calling 911, placing the overdose victim in the recovery position, etc.). All but one study discussed overdose recognition (McAuley et al. 2010). Program duration ranged from around 13 to 90 min. Three studies did not report program duration (Lankenau et al. 2013; McAuley et al. 2010; Strang et al. 2008).

Many studies neglected to report the curriculum models and evaluation tools used to develop these programs. Therefore, although we can determine that content was similar, other aspects of the training sessions, such as training methods for delivering the content, the duration of the session, and evaluation protocol may have differed across programs. Of the four studies that did name papers or organizations which influenced the development of the training curriculum, there was no overlap among curriculum models. Regarding evaluation tools, the Brief Overdose Recognition and Response Assessment (BORRA) scale (Green et al. 2006) was referenced by two studies. Williams et al. (2014) cited their own scales. The other four studies discussed questionnaire and interview assessment items but did not include citations (Gaston et al. 2009; Strang et al. 2008; Lankenau et al. 2013; McAuley et al. 2010; Galea et al. 2006).

#### Quantitative results

Summing across all four studies of naloxone lay administration effectiveness, a total of 66 witnessed overdose events were reported. Of the 66 witnessed events, 39 (59.1%) recovered after naloxone was administered by a lay participant and 22 (33.3%) recovered without the administration of naloxone. There were no deaths among the 39 instances when naloxone was administered. There were three deaths among the 27 instances when naloxone was not administered, for an 11.1% mortality rate. The outcome of two witnessed events (3%) was unknown.

**Figure 2:**
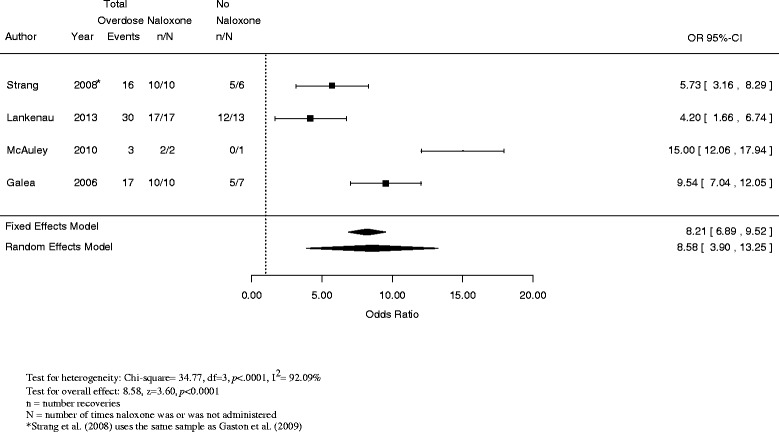
Estimated odds ratios of recovery from drug overdose associated with naloxone administration by bystanders.

For the four studies of naloxone lay administration effectiveness (Strang et al. 2008; Lankenau et al. 2013; McAuley et al. 2010; Galea et al. 2006), the summary odds ratio measuring the strength of the association between naloxone administration and recovery based on the data reported was 8.58 (95% confidence interval (CI) = 3.90 to 13.25, *I*^2^ = 92.09%), indicating a statistically significant but highly heterogeneous effect (see Figure 2).

**Figure 3:**
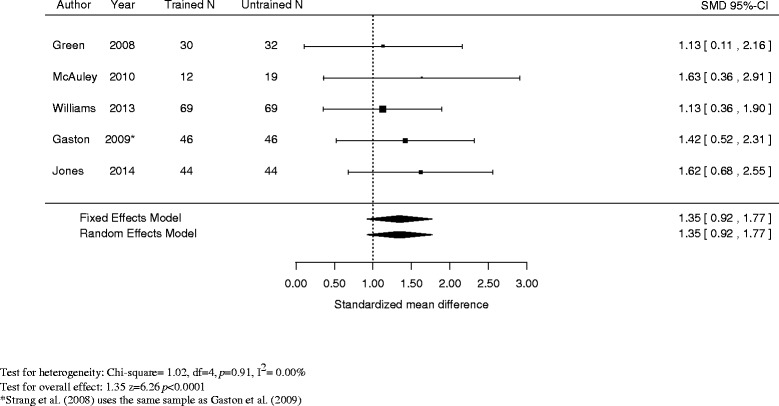
Mean difference in training knowledge score between trained and untrained participants.

Of the five studies that reported training outcomes (Green et al. 2008; McAuley et al. 2010; Williams et al. 2014; Gaston et al. 2009; Jones et al. 2014) (see Figure 3), the overall average scores were significantly higher for trained participants than untrained participants on tests that covered overdose prevention material (naloxone administration, overdose recognition, overdose response) (standardized mean difference = 1.35, 95% CI = 0.92 to 1.77, *I*^2^ = 0.00%).

### Discussion

Lay administration of naloxone is increasingly being used as a response to the epidemic of opioid-related fatality in the United States. In this review, we found that such administration was both safe and effective and that these programs are equipped to train participants in overdose management protocol.

A systematic review of community opioid overdose prevention and naloxone distribution programs was recently published (Clark et al. 2014), and qualitative data has been summarized (CDC 2012). Our study adds to these results by synthesizing quantitative findings on the effectiveness of bystander naloxone distribution and overdose education. Our findings offer support for the World Health Organization’s consensus report in favor of naloxone distribution and gives further reason to proceed with the guidelines developed by their report, which include expanding the availability of naloxone to lay people (WHO 2014).

Both of the analyses in our study favor the treatment, overdose prevention training, and lay naloxone administration. Our findings imply that lay-dispensed naloxone is effective in treating overdose and that training has been successful in improving participant knowledge of overdose recognition and management.

The results imply that such programs are equipped to train non-medical community members to respond to overdose events. As previous studies have suggested, lay community members can be trusted to safely and adequately dispense naloxone to victims. By combining the results of small-N studies, these meta-analyses disambiguate the literature on these programs.

Although empirical findings support naloxone distribution, legal barriers at the state level have stood in the way of implementation of these programs across the nation. Further research should compare the success of programs in states which have removed these legal barriers to the success of programs in states where these barriers remain. Although many states have updated their laws to allow naloxone distribution, there are still programs operating in states without any naloxone access laws (Law Atlas 2014). There are also programs in states which permit naloxone distribution but have yet to adopt a 911 Good Samaritan law, which gives legal protection to non-medical individuals who provide emergency assistance (Davis 2014). It is crucial to assess how overdose prevention programs operate in these climates in order to determine the effects of legal barriers on the success of naloxone distribution and overdose education programs across the nation.

#### Limitations

There was substantial heterogeneity in the meta-analysis of lay administration of naloxone. The high heterogeneity between studies is likely due to the wide range of total overdose events reported among the four studies. For the training outcome, the participant population was the same for all but one study, which observed family and caregivers of users instead of opioid users themselves (Williams et al. 2014). In addition, some studies only included responses regarding naloxone administration into the calculation of mean scores. Other studies pooled responses on naloxone administration with responses on other material, such as overdose recognition and response. Finally, the majority of the participants in these studies were self-identified heroin users or their families and peers, without medical training. Therefore, results from this meta-analysis may not be directly generalizable to opioid users or healthcare practitioners. Despite these limitations, this study provides a much-needed estimate of the benefits and risks associated with lay administration.

## Conclusions

In conclusion, our findings support overdose education and lay administration of naloxone as a safe and effective community-based approach to controlling the opioid overdose epidemic. Both meta-analyses favor the treatment, suggesting that lay naloxone administration and overdose training are associated with increased odds of recovery and increased knowledge of overdose recognition and management. These findings can inform policy decisions as policy-makers consider whether or not to expand these initiatives.
